# High-dose intravenous methylprednisolone for the prophylactic treatment of cluster headache

**DOI:** 10.1186/2193-1801-2-156

**Published:** 2013-04-11

**Authors:** Sanami Kawada, Kenichi Kashihara, Takaki Imamura, Manabu Ohno

**Affiliations:** Department of Neurology, Okayama Kyokuto Hospital, 567-1 Kurata, Naka-ku, Okayama, 703-8265 Japan

**Keywords:** Cluster headache, Methylprednisolone, Steroid pulse therapy

## Abstract

**Background:**

Triptans are effective for immediate relief of episodic cluster headache (CH) but do not reduce the frequency of attacks. Intravenous bolus injection of corticosteroids like methylprednisolone (MP) has been reported to decrease the frequency of CH attacks. We validated the prophylactic efficacy of MP pulse therapy by monitoring CH recurrence over several years following treatment of six consecutive male patients (mean age: 38.8 years, range: 26–54 years) afflicted by frequent (often daily) CH attacks.

**Findings:**

Total MP dose per infusion was 250–500 mg for five patients and 125 mg for the sixth (a diabetic). High-dose MP was administered for 2 or 3 consecutive days in hospital for the first two patients treated. The next four patients received a single bolus injection at presentation, and in some cases a second injection days later at an outpatient clinic. The first two cases treated were also prescribed daily oral prednisolone for at most 6 months while the latter four cases were not. The frequency of CH attacks was markedly reduced in all patients, with intervals between attacks ranging from 4 to 23 months. We noted no apparent adverse events following MP administration.

**Conclusions:**

High-dose MP therapy reduced CH attack frequency and improved patient quality of life.

## Introduction

Subcutaneous injection of sumatriptan succinate is an effective treatment for the immediate relief of episodic cluster headache (CH) but does not reduce the frequency of future attacks. Prolonged or frequent attacks and associated treatment visits can greatly impair patient quality of life. In Japan, self-administration of sumatriptan succinate was approved in February 2008, and this change has greatly reduced emergency visits and hospital admissions due to CH. However, patients with chronic CH may still require frequent hospital visits, taxing resources and increasing personal medical expenses. To reduce the frequency of attacks, guidelines from the Japanese Society of Neurology recommend calcium channel blockers (verapamil, lomerizine hydrochloride), glucocorticoids, or ergotamine tartrate. Calcium channel blockers are often useful in the prevention of migraine headache, but are less effective in the prevention of cluster headaches. Oral glucocorticoids are often effective against CH: however, the side effects may be intolerable for some patients. Several recent studies have reported the benefits of intravenous bolus injection of methylprednisolone (MP) for episodic CH. In this case series, we evaluated the efficacy of this treatment for Japanese male patients experiencing frequent CH attacks.

## Material and methods

This case series reports the clinical results for six consecutive male patients with frequent episodic CH treated by bolus injection of MP. MP was administered by intravenous (i.v.) drip over two hours up to a total dose of 250–500 mg per infusion with the exception of a diabetic patient who was administered 125 mg MP per infusion. If the patient experienced a severe CH attack at presentation, they were injected subcutaneously with sumatriptan succinate before MP injection. The first two cases in the series were admitted to hospital and received MP pulse therapy for 2 or 3 consecutive days as inpatients. If CH was not suppressed, a second course of pulse therapy was administered after a four-day interval. The latter four patients could not be admitted due to work or domestic circumstances, and thus were administered a single bolus MP injection and in some cases, a second bolus injection at a later time in an outpatient department (Table 1). This study was approved by The Ethics Committee of Okayama Kyokuto Hospital and informed consent was obtained from all subjects prior to the study.

**Table 1:** **Cluster headache attacks and steroid theraphy**

Case	Age	Sex	Intervals between CH attacks (months)	Methylprednisolone dose (intravenous)	Admission	Prednisolone dose and duration (daily oral)
1	54	Male	Initial presentation	250 mg (Days 1 and 2)	Yes	10 mg, 90 days
			4	250 mg (Days 1–3)	Yes	none
			4	250 mg (Days 1 and 2)	Yes	5 mg, 60 days
			5	250 mg (Day 1)	No	5 mg, 180 days
2	33	Male	Initial presentation	250 mg (Days 1–3, 7–9)	Yes	20 mg, 10 days
			23	250 mg (Days 1 and 9) + 500 mg (Day 4)	No	15 mg, 30 days
			11	500 mg (Days 1, 7, 11, and 13)	No	10 mg, 40 days
3	33	Male	Initial presentation	250 mg (Day 1) + 500 mg (Day 18)	No	none
			6	250 mg (Day 1)	No	none
4	38	Male	Initial presentation	250 mg (Day 1)	No	none
			11	250 mg (Day 1)	No	none
			7	250 mg (Day 1)	No	none
			5	250 mg (Day 1)	No	none
			7	250 mg (Day 1)	No	none
5	49	Male	Initial presentation	125 mg (Days 1 and 44)	No	none
			7	125 mg (Day 1)	No	none
6	26	Male	Initial presentation	500 mg (Day 1)	No	none
			11	500 mg (Day 1)	No	none

Day 1: the 1^st^ day of MP injection during one CH attack.

## Results

At presentation, all six patients reported daily CH attacks that required subcutaneous injection of sumatriptan succinate and (or) inhalation of pure oxygen. These therapies were effective, but the frequent attacks seriously disrupted quality of life. The MP pulse therapy regimens used on initial presentation, the times of recurrence, and oral prednisolone regimens are presented in Table. In all cases, CH attack frequency was markedly reduced following MP therapy and no patient reported severe CH for at least four months after treatment. Case 1 relapsed thrice and was treated as an inpatient except on the fourth occasion. Case 2 reported no headache for two days after the first trial of MP pulse administration (one injection per day for three consecutive days): however, he suffered from severe headache again on the sixth day of admission and was administered another series of injections. Case 2 relapsed only twice, although 3 or 4 non-consecutive high-dose bolus injections were required on each recurrence. The other four cases were never hospitalized. Three of these four patients relapsed only once (6, 7, and 11 months after initial treatment) and were treated by a single bolus injection of MP each time. Case 4 relapsed four times, but also required only a single bolus injection each time, and each treatment required only a half-day absence from work. We noted no adverse events following steroid pulse therapy, such as gastrointestinal bleeding or psychiatric symptoms, and the diabetic patient (Case 5) demonstrated no loss of glucose control.

## Discussion

Cluster headaches afflict 0.07-0. 09% of the population, and the incidence is 9.8 events/million person-years Swanson et al. (1994). The prevalence of CH is 5–9 times higher in men, and indeed all patients in this case series were men. Cluster headache is a rare disease but symptom severity precludes placebo-controlled studies. Therefore, therapeutic regimens are inconsistent across institutions and countries. According to the Japanese guidelines for chronic headache, triptans or oxygen inhalation is the first-line therapy for acute CH attacks, and most patients respond well to either treatment. However, it is recommended that inhalation of 100% oxygen be initiated within 10 minutes of attack onset, making this treatment option impractical for patients with unpredictable CH. Subcutaneous sumatriptan injection is effective and Japanese approval of self-injection (December 2007) has reduced hospital visits due to CH. Intranasal sumatriptan and oral zolmitriptan (intranasal zolmitriptan is not yet approved in Japan) are also effective for patients that cannot self-inject sumatriptan. Intranasal lidocaine or cocaine is also effective but rarely used in Japan.

During active CH periods, severe headaches occur almost daily, interfering with work or school and presenting patients with a significant financial burden for drugs or oxygen. Prophylactic therapy would thus greatly improve quality of life, relieve financial hardship, and save medical resources. The guidelines from the Japanese Society of Neurology recommend ergotamine, calcium-channel blockers, or corticosteroids for prophylaxis at the beginning of a cluster period, although the efficacy of these treatments is highly variable. Double-blind trials have demonstrated the effectiveness of verapamil, but bradycardia and heart block are serious risks. Although lomerizine has less severe cardiovascular side effects and is useful for migraine, there is currently no evidence that lomerizine is an effective prophylactic treatment for CH. Moreover, there are no double-blind placebo-controlled studies of corticosteroid efficacy: nonetheless, corticosteroids have been used for rapid CH control for more than 60 years Horton (1952). Oral and intravenous corticosteroids may be beneficial Ashkenazi & Schwedt (2011); Shapiro (2005). For oral administration, daily 40–80 mg prednisolone with gradual reduction over 10 days or longer is recommended Couch & Ziegler (1978).

There are few reports on the efficacy of intravenous steroid infusion Reija et al. (1998); Cianchetti et al. (1998); Mir et al. (2003); Antonaci et al. (2005). Reija et al. (1998) reported a series of 18 episodic CH patients treated with 16 mg dexamethasone (i.v.) tapering off after 15 days. On this cohort, 17 patients showed reduced CH attack frequency Reija et al. (1998). Cianchetti et al. (1998) reported a single case with chronic CH who showed no response to two weeks of daily 100 mg prednisone but responded dramatically to single 500 mg doses of intravenous MP Cianchetti et al. (1998). Mir et al. (2003) treated 14 episodic CH patients with 250 mg intravenous MP on three consecutive days, followed by 90 mg oral prednisone with gradual tapering over 4 weeks, and reported superior results compared to previous treatments Mir et al. (2003). Finally, Antonaci et al. (2005) treated 13 episodic CH patients with 30 mg/kg intravenous MP on the eighth day of the active CH period and reported significantly reduced attack frequency in all patients, whereas three patients experienced complete headache remission Antonaci et al. (2005).

The therapeutic benefits of corticosteroids may stem, at least in part, from anti-inflammatory effects because inflammation may play an important role in the onset and exacerbation of CH Hannerz (1991). Orbital phlebography and gallium single photon emission computerized tomography revealed inflammation in the cavernous sinus ipsilateral to the CH pain side during the attack Gawel et al. (1990); Hardebo & Ryberg (1989). Moreover, elevated serum concentrations of some inflammatory mediators such as haptoglobin, orosomucoid, and interleukin-I beta, have been reported during CH attacks Hannerz (1991); Martelletti et al. (1993). Several investigations have reported elevated baseline plasma cortisol levels during CH attacks Waldenlind et al. (1987); Leone et al. (1995); Strittmatter et al. (1996). In addition to cortisol, testosterone and melatonin may also be linked to the pathogenesis of CH Stillman (2006). Positron emission tomography revealed hyperactivation in the gray matter of the ipsilateral hypothalamus during the acute pain state May et al. (1998); Sprenger et al. (2004). Hypothalamic activation coupled with elevated serum cortisol suggests that episodic CH patients may have a dysfunctional hypothalamic-pituitary-adrenal axis. Recently, hypothalamic deep brain stimulation (hDBS) has been used effectively to treat CH patients Magis et al. (2012). The therapeutic mechanism of hDBS may be similar to high-dose MP injection, suppression of hypothalamic hyperactivity by negative feedback.

High-dose MP injection may produce serious side effects, including infectious diseases resulting from immunosuppression, hypertension, and elevated blood glucose: therefore, this treatment option should be used with caution (particularly as outpatient therapy) in the elderly or for patients with serious underlying diseases such as diabetes. However, MP is far less invasive than hDBS and so should be considered before hDBS. Moreover, younger patients and those without serious contraindication rarely exhibit serious side effects in response to high-dose MP. Finally, MP pulse therapy can be administered rapidly with little need for school or work absence. Thus, high-dose MP injection may be an optimal treatment for otherwise healthy active CH patients.
